# Microphthalmia and Infantile Spasms Leading to the Diagnosis of Aicardi Syndrome: A Case Report and Literature Review of a Rare Entity

**DOI:** 10.7759/cureus.99822

**Published:** 2025-12-22

**Authors:** M'hamed Riad Amanallah, Mohamed Chraa, Nissrine Louhab, Khaoula Balili

**Affiliations:** 1 Neurology, Mohammed VI University Hospital, Faculty of Medicine and Pharmacy, Cadi Ayyad University, Marrakech, MAR

**Keywords:** agenesis of corpus callosum, aicardi syndrome, chorioretinal lacunae, hypsarrhythmia, infantile spasms, microphthalmia

## Abstract

Aicardi syndrome is a rare neurodevelopmental disorder characterized by the triad of corpus callosum agenesis, chorioretinal lacunae, and infantile spasms. Clinical presentation may vary widely, and ocular abnormalities can precede neurological symptoms. We report a case of a four-month-old female infant, initially referred for evaluation of unilateral microphthalmia and mild developmental delay. Ophthalmological examination revealed marked right microphthalmia, chorioretinal lacunae, and an optic disc coloboma in the left eye. Approximately one month later, she presented with new-onset, clustered episodes of axial flexion spasms, characteristic of infantile spasms. An electroencephalogram showed hypsarrhythmia, leading to a diagnosis of West syndrome. Subsequent brain magnetic resonance imaging revealed complete agenesis of the corpus callosum, multiple interhemispheric and intraventricular cysts, and a posterior fossa cyst. The constellation of these findings confirmed the diagnosis of Aicardi syndrome. The definitive diagnosis relies on integrating ophthalmological findings with neuroimaging and electrophysiological studies. Early recognition of the triad, especially when atypical ocular features such as unilateral severe microphthalmia act as an early presenting feature preceding the onset of spasms, allows for prompt initiation of anti-epileptic therapy and coordinated multidisciplinary care, which is essential for managing the severe developmental and neurological challenges associated with this rare syndrome.

## Introduction

Aicardi syndrome (AS) is a rare genetic neurodevelopmental disease characterized by a classic triad: complete or partial agenesis of the corpus callosum, chorioretinal lacunae, and infantile spasms. The condition is reported almost exclusively in girls and presents in early infancy. It is believed to result from a de novo mutation on the X chromosome. Despite its genetic origin, all known cases of AS have been sporadic with no specific causative gene identified [1].

With an estimated incidence of approximately one in 105,000 to 167,000 live births, the phenotypic spectrum of Aicardi syndrome is broad with variable clinical presentations. The hallmark of the disease is infantile spasms. However, other signs such as microphthalmia, structural eye abnormalities, and developmental delay may precede the full clinical picture and raise suspicion for the diagnosis. Neuroimaging and ophthalmologic findings play a key role in diagnosis, often revealing additional features such as cortical malformations, intracranial cysts, and colobomas [2,3].

This case is reported to emphasize the diagnostic value of early ocular abnormalities in raising suspicion for Aicardi syndrome and to discuss this rare neurodevelopmental entity.

## Case presentation

A four-month-old female infant, the first child of a healthy, non-consanguineous couple with no relevant family history of neurological or genetic disorders, was referred to our neurology department for evaluation of new-onset abnormal movements occurring multiple times a day. The patient had no significant prenatal or perinatal complications. Her psychomotor evaluation revealed a slight delay, with reduced visual tracking and poor head control noted by the mother at around three months of age.

Initially, the infant was seen by an ophthalmologist after parental concern regarding asymmetry of the eyes. Examination revealed marked microphthalmia of the right eye, preventing detailed fundus evaluation. Fundoscopy of the left eye showed multiple chorioretinal lacunae in the posterior pole and mid-periphery, surrounded by pigmentary changes, along with an optic disc coloboma. These findings raised suspicion for an underlying syndromic etiology, though no definitive diagnosis could be made at that time.

Approximately one month later, in August 2024, the child presented to our department for further evaluation. The parents reported clustered episodes of axial flexion of the neck and trunk, associated with limb movements, occurring up to 50 times per day, typical of infantile spasms. On examination, the infant appeared irritable, had limited visual engagement, and demonstrated axial hypotonia. Head circumference was appropriate for age, with no dysmorphic features or organomegaly.

**Figure 1:**
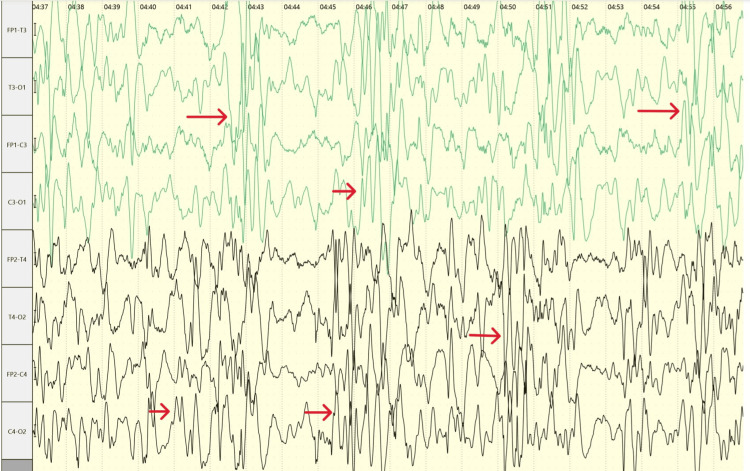
EEG showing a hypsarrhythmia pattern characteristic of infantile spasms in Aicardi syndrome.

An electroencephalogram (EEG) was performed and revealed a chaotic, high-amplitude background with multifocal spikes and slow waves consistent with hypsarrhythmia (Figure 1). Based on the clinical and EEG findings, a diagnosis of West syndrome was made. The patient was immediately initiated on vigabatrin at a dose of 50 mg/kg/day.

**Figure 2:**
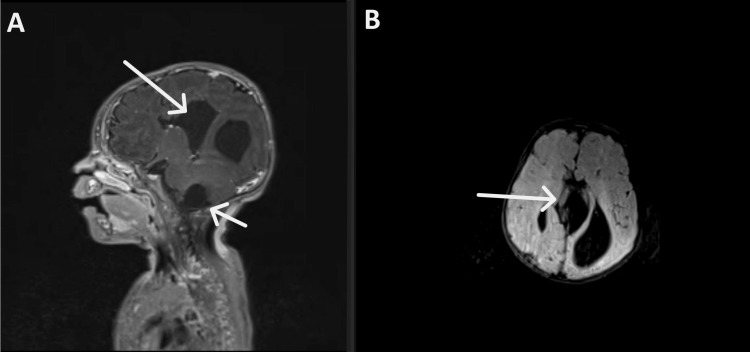
(A) Sagittal T1-weighted post-contrast MRI showing an interhemispheric cyst and a cyst in the posterior part of the medulla oblongata. (B) Axial T2-FLAIR (fluid-attenuated inversion recovery) MRI demonstrating the interhemispheric cyst and agenesis of the corpus callosum.

To further evaluate the etiology of the spasms and the constellation of ocular findings, a brain magnetic resonance imaging (MRI) was ordered. The MRI demonstrated agenesis of the corpus callosum along with interhemispheric cysts most prominent around the midline, an intraventricular cyst involving the choroid plexus of the left lateral ventricle, and a cyst in the posterior part of the medulla oblongata responsible for a dilation of the fourth ventricle (Figure 2). These radiological findings, in conjunction with the microphthalmia, chorioretinal lacunae, optic disc coloboma, infantile spasms, and hypsarrhythmia, were diagnostic of Aicardi syndrome.

Following the diagnosis, the family was counseled regarding the nature of the disease, its genetic implications, and the expected clinical course. The patient was referred for ongoing multidisciplinary care, including pediatric neurology, ophthalmology, and early developmental intervention services. Genetic testing was not performed, as Aicardi syndrome currently lacks an identifiable causative gene and remains a clinical diagnosis. This was clearly explained to the family.

At six months follow-up after treatment initiation, the frequency of spasms had decreased, developmental delay persisted, and ophthalmologic findings remained stable. She continues to be monitored by neurology and ophthalmology teams.

## Discussion

Aicardi syndrome is thought to be X-linked dominant and male-lethal, as it affects almost exclusively females and very rarely XXY males, and typically arises de novo. Aicardi syndrome remains genetically heterogeneous, with no single gene mutation consistently identified; thus, the syndrome remains genetically enigmatic. The most recently published genetic studies of AS are now utilizing next-generation sequencing technologies for the generation and analysis of genomic and transcriptomic level data. A limited number of individuals with AS have undergone sequencing and subsequent analyses [4,5].

Some studies suggest that Aicardi syndrome may not be restricted to X-linked genes, and that autosomal mutations like TEAD1 may underlie certain cases. A recent study conducted on 10 females with confirmed or suspected Aicardi syndrome suggests that it may not be a monogenic X-linked disorder, shifting the paradigm toward a multi-gene, de novo model involving autosomal genes crucial for cortical formation. The study emphasizes the involvement of genes linking to cortical development pathways and chromatin regulation, including Wnt signaling (WNT8B) and transcriptional/chromatin modulators (KMT2B, SMARCB1, SZT2); however, larger cohorts and deeper sequencing are needed to validate these findings [6,7].

Aicardi syndrome is rare and might be underdiagnosed. The infantile spasms and agenesis of the corpus callosum, which constitute a part of the triad, are nonspecific and can be associated with various underlying disorders, both genetic and non-genetic. The triad of chorioretinal lacunae, infantile spasms, and agenesis of the corpus callosum is almost pathognomonic for the condition [8].

Infantile spasms are the most characteristic seizure type, typically starting around the age of three to four months. They are often asymmetric, presenting in the form of clusters of axial flexor or extensor spasms. Over time, medically refractory epilepsy may develop with both focal and generalized seizure types (Lennox-Gastaut syndrome pattern) [1,4,8].

The typical electroencephalography (EEG) findings are asynchronous multifocal epileptiform discharges, consistent with a burst suppression pattern and interhemispheric dissociation. Typically, the tracing is marked by intermittent bursts of high-amplitude slow and sharp waves, separated by periods of low-voltage or even absent electrical activity. This burst-suppression pattern is usually asymmetric. Hypsarrhythmia, such as in our patient, which is defined as a pattern of high-voltage arrhythmic slow waves interspersed with spike discharges, can also be found less frequently, with an incidence ranging between 29% and 36% in some studies [9,10]. An EEG pattern of hypsarrhythmia may predict a less severe motor and language outcome compared to burst-suppression [9].

Neuroimaging plays a key role in the diagnosis of Aicardi syndrome. An MRI must be performed in any patient with infantile spasms as an etiologic work-up [11], with characteristic findings that support clinical suspicion. In our patient, brain MRI revealed complete agenesis of the corpus callosum, which is a hallmark feature of the syndrome, along with interhemispheric cysts, an intraventricular choroid plexus cyst, and a posterior fossa cyst causing fourth ventricle dilation, a constellation of findings consistent with the known radiological spectrum of the disease [12].

Corpus callosum agenesis is one of the most consistent neuroanatomical abnormalities and is an inclusion criterion for the diagnosis. The absence of the corpus callosum contributes to the typical interhemispheric EEG dissociation and plays a role in the neurological manifestations. However, rare cases of normal corpus callosum and/or partial agenesis have been reported, emphasizing the importance of other neuronal migration abnormalities for the diagnosis and how any future discovery of causative genes may help include patients with mild forms of the disease [12].

Other brain manifestations include subcortical and periventricular heterotopias, choroid plexus papillomas, cerebellar dysmorphia, polymicrogyria, and posterior fossa and interhemispheric cysts [1].

Intracranial cysts are characteristic. In a study of 23 individuals with Aicardi syndrome, cerebral cysts were seen in 95% of cases and are typically midline/paramedian or intraventricular. These cysts are often non-communicating and may distort adjacent structures, and may show contrast enhancement of the cyst wall [12]. Our patient had prominent cysts near the midline, consistent with the classic imaging profile. While posterior fossa anomalies are not as frequently emphasized in the Aicardi literature, malformations of the brainstem and cerebellum, including cystic changes or hypoplasia, can be seen and are probably underestimated, as they were observed in the majority of patients in the same study of 23 patients. Choroid plexus papillomas and choroid plexus cysts are characteristic as well, as they have been described frequently [13].

The most common eye abnormalities in Aicardi syndrome are chorioretinal lacunae, found in the large majority of patients with the condition and considered pathognomonic. They are yellow or yellowish white in appearance, round in shape, variable in size, and they do not change or evolve over time. As long as the causal mechanism of the syndrome is not elucidated, chorioretinal lacunae are considered essential for the clinical diagnosis. Optic nerve abnormalities are represented by the optic nerve coloboma as the most frequent abnormality. Microphthalmia can also be seen, and, if severe enough, it can interfere with the ophthalmological examination [2,4].

Extracranial anomalies, such as rib or vertebral defects and scoliosis, occur in about half of patients [1]. Tumors are rare, with choroid plexus papillomas being most frequently reported [4,14].

Aicardi syndrome typically manifests with severe neurodevelopmental impairment, with most individuals displaying significant intellectual disability [15]. The prognosis is guarded, as many girls die of complications of their syndrome at a young age. The spectrum of severity may allow some patients to live into their adolescent years and 20s [16].

## Conclusions

Aicardi syndrome is a rare neurodevelopmental disorder that requires a high index of suspicion for diagnosis. In this case, neuroimaging revealed complete agenesis of the corpus callosum, interhemispheric and intraventricular cysts, and a posterior fossa cyst, while EEG confirmed hypsarrhythmia, completing the classical triad and allowing a definitive diagnosis. Early recognition of atypical ocular abnormalities, such as unilateral microphthalmia and chorioretinal lacunae, in conjunction with neuroimaging and electrophysiological studies, facilitated prompt diagnosis and initiation of treatment. This case underscores the importance of a multidisciplinary approach involving pediatric neurology, ophthalmology, genetics, and rehabilitation services to optimize management and potentially improve long-term outcomes.
